# C2α‐carbanion‐protonating glutamate discloses tradeoffs between substrate accommodation and reaction rate in actinobacterial 2‐hydroxyacyl‐CoA lyase

**DOI:** 10.1002/2211-5463.70199

**Published:** 2026-01-28

**Authors:** Michael Zahn, Barbara Seroka, Ryszard Lazny, Zenon Lotowski, Thore Rohwerder

**Affiliations:** ^1^ Centre for Enzyme Innovation, School of the Environment and Life Sciences University of Portsmouth UK; ^2^ Core Facility Protein Production, Biozentrum, Martin‐Luther‐University‐Halle‐Wittenberg Germany; ^3^ Faculty of Chemistry University of Bialystok Poland; ^4^ Department of Microbial Biotechnology, Helmholtz Centre for Environmental Research – UFZ Leipzig Germany

**Keywords:** 2‐hydroxyacid synthesis, biotechnology, building block chemicals, carbon fixation, pyruvate oxidase family, synthetic biology

## Abstract

Thiamine‐dependent actinobacterial 2‐hydroxyacyl‐CoA lyase (AcHACL) catalyzes the reversible cleavage of 2‐hydroxyacyl‐CoAs to formyl‐CoA and carbonyl compounds. To exploit the enzyme's biotechnological potential, a deeper understanding of the catalysis is required. Previously, AcHACL E493 was identified as an important acid/base catalyst. Here, wild‐type and E493 mutant crystal structures representing Michaelis complexes with 2‐hydroxyisobutyryl‐CoA and (*S*)‐2‐methylglyceryl‐CoA are provided. Although E493 guarantees high rates of essential proton transfers in AcAHCL‐catalyzed on‐pathway cleavage of 2‐hydroxyacyl‐CoAs and off‐pathway carboligations with short‐chain aldehydes and ketones, wild‐type substrate accommodation is suboptimal. Not E493D, but E493A and E493S mutations improved *K*_M_. However, *k*_cat_ is substantially reduced in the mutants. These tradeoffs are discussed by comparing active sites of AcHACL and related enzymes either lacking or possessing an E493 homolog.

## Graphical abstract

Enzymes of the 2‐hydroxyacyl‐CoA lyase group catalyze the condensation of formyl‐CoA with aldehydes or ketones. Thus, by structural adaptation of active sites, practically any pharmaceutically and industrially important 2‐hydroxyacid could be biotechnologically synthesized. Combining crystal structure analysis, active site mutations and kinetic assays, we show that an actinobacterial lyase has great potential for the stereospecific synthesis of short‐chain aliphatic 2‐hydroxyacids.

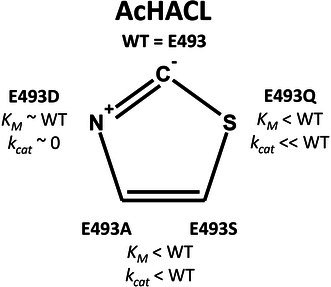

**Abbreviations**
2‐HIB: 2‐hydroxyisobutyryl
AcHACL: actinobacterial 2‐hydroxyacyl‐CoA lyase
AHAS: aceto‐2‐hydroxyacid synthase
dzThDP: 3‐deazathiamine diphosphate
HACL: 2‐hydroxyacyl‐CoA lyase
OXC: oxalyl‐CoA decarboxylase
PDC: pyruvate decarboxylase
POX: pyruvate oxidase
SAAT: sulfoacetaldehyde acetyltransferase
ThDP: thiamine diphosphate
WT: wild‐type

## Introduction

Thiamine diphosphate (ThDP)‐dependent enzymes catalyze a broad range of reactions, such as electron transfer, decarboxylation, and reversible covalent bond formations [1]. They are involved in central metabolic pathways, for example, pyruvate dehydrogenase catalyzes the NADH‐forming oxidative decarboxylation of pyruvate to acetyl‐CoA. Alternatively, pyruvate can be decarboxylated to acetyl‐P by pyruvate oxidase (POX) or to acetaldehyde by pyruvate decarboxylase (PDC). The biosynthesis of branched‐chain amino acids, on the other hand, is initiated by aceto‐2‐hydroxyacid synthase (AHAS), producing acetolactate from the condensation of two pyruvate molecules. Moreover, carbonyl compound condensations hold great potential in biotechnology, for example, for the design of one‐carbon assimilation routes [2] or for the synthesis of chiral tertiary alcohols [3] and 2‐hydroxy‐3‐oxoacids [4].

**Fig. 1:**
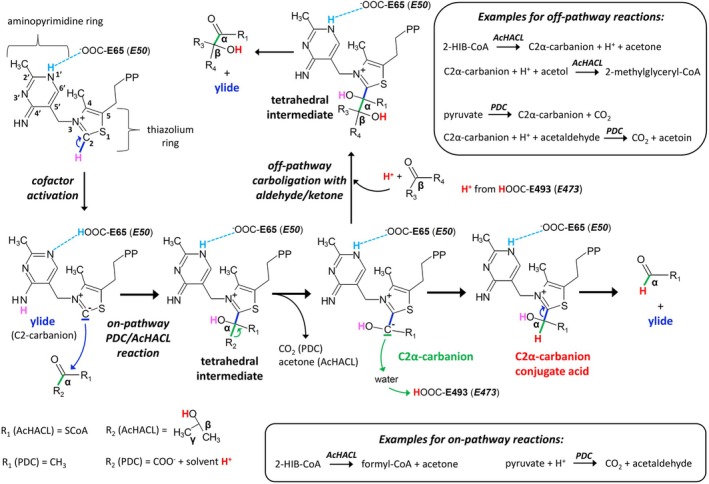
Proposed proton transfers in ThDP‐dependent reactions catalyzed by bacterial PDC (UniProt ID: P06672) and actinobacterial 2‐hydroxyacyl‐CoA lyase (AcHACL, UniProt ID: P0DUV9). Catalytic Glu residues involved in cofactor activation and C2α‐carbanion protonation are indicated (numbering as in AcHACL and in parenthesis for bacterial PDC). While the cofactor‐activating Glu is conserved in almost all ThDP‐dependent enzymes, the C2α‐carbanion‐protonating residue can only be found in a few enzymes (Table 1). In the AcHACL‐catalyzed reaction, the proton of the substrate acyl‐Cβ‐OH group is transferred to the C2α‐carbanion (highlighted in red in leaving group *R*_2_). In contrast, the proton is provided from solvent in the PDC‐catalyzed decarboxylation reaction. Stoichiometry of the PDC‐catalyzed off‐pathway acetoin formation from pyruvate requires one complete on‐pathway reaction cycle to acetaldehyde and a second incomplete cycle to the C2α‐carbanion. In practice, acetoin formation from pyruvate as donor substrate is enhanced by exogenous acetaldehyde as acceptor substrate [7]. Dashed lines indicate conserved H bonds in ThDP‐dependent enzymes. Reaction arrows are only shown for the forward reactions.

**Fig. 2:**
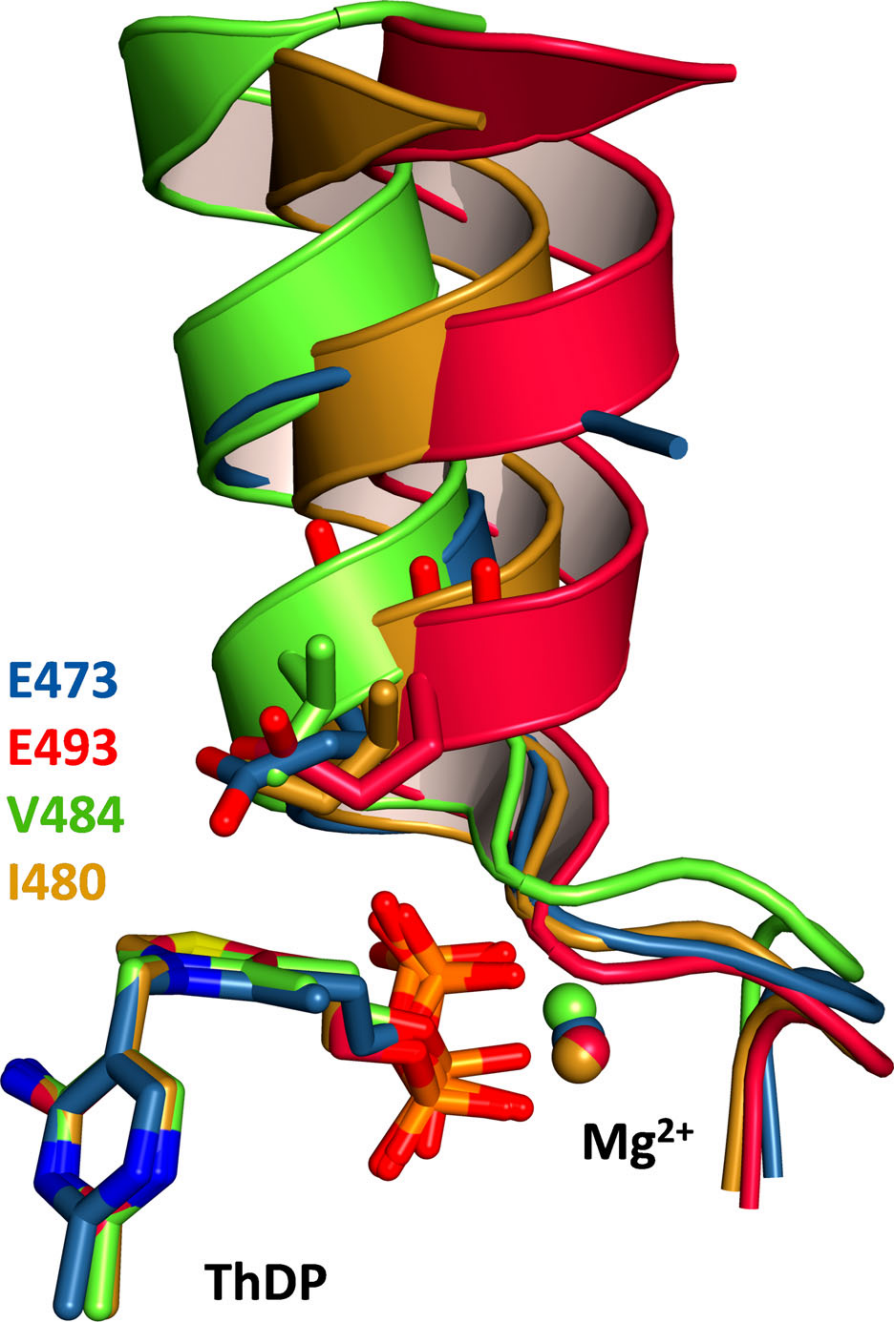
Positioning of C2α‐carbanion‐protonating Glu perpendicular above the ThDP cofactor thiazolium ring in the POX family of ThDP‐dependent enzymes. In bacterial PDC (E473, PDB ID: 2WVA, blue) and AcHACL (E493, PDB ID: 7PT1, red), the catalytic residue is located on a small α‐helix directly following the conserved loop which is involved in Mg^2+^ binding. The α‐helix possesses one turn in bacterial PDC and three turns in AcHACL. The three‐turn helix but not the C2α‐carbanion‐protonating Glu is conserved in bacterial AHAS (V484, PDB ID: 4RJI, green) and POX (I480, PDB ID: 4KGD, orange).

A hallmark of these enzymes is the highly conserved proton transfer involved in cofactor activation and catalysis [5]. In almost all ThDP‐dependent enzymes, the interaction of a catalytic Glu (E65 in Fig. 1) with the N1′ atom of the aminopyrimidine ring induces amino‐imino tautomerism, thus enabling deprotonation of the thiazolium C2 atom. The resulting ylide (C2‐carbanion) is a reactive nucleophile and can attack the electrophilic center in a carbonyl compound (donor). During this attack, the substrate carbonyl‐O atom is protonated by cofactor N4′ and the ThDP‐bound tetrahedral intermediate is formed. In case of POX, PDC, and AHAS, the reaction proceeds via decarboxylation to the C2α‐carbanion as central nucleophilic intermediate. The latter attacks another carbonyl compound (acceptor) in the AHAS‐catalyzed carboligation and transfers its free electron pair to FAD in POX. In contrast, the C2α‐carbanion is protonated in the PDC reaction to the conjugate acid, from which acetaldehyde is finally released. This proton transfer is likely mediated by another catalytic Glu [6] conserved in bacterial (E473, UniProt ID: P06672) as well as yeast PDC (E477, UniProt ID: P06169). Interestingly, yeast PDC on‐pathway conversion of pyruvate to acetaldehyde is dramatically reduced by mutation E477Q, whereas off‐pathway carboligation with pyruvate (donor) and acetaldehyde (acceptor) to acetoin (Fig. 1) becomes the dominant reaction [7]. This indicates that C2α‐carbanion protonation is a crucial step in PDC catalysis. In the structures of bacterial (PDB ID: 2WVA) and yeast (PDB ID: 1PVD) PDC, the E473/E477 residue is directly positioned perpendicular above the cofactor thiazolium ring. It is part of the first turn of a small α‐helix located at the entrance to the active site (Fig. 2). This α‐helix is conserved in most members of the POX enzyme family (including PDC, AHAS, and POX). However, the PDC E473/E477 is often replaced by a hydrophobic residue, such as Leu, Val or Ile, in other enzymes (Table 1).

**Table 1:** On‐pathway reactions catalyzed by POX family enzymes.

Enzyme	Reaction	AcHACL E493 homolog	AcHACL Q128 homolog	UniProt entry
**With on‐pathway protonation of C2α‐carbanion/deprotonation of its conjugate acid**				
Actinobacterial 2‐hydroxyacyl‐CoA lyase (AcHACL)	2‐hydroxyacyl‐CoA → aldehyde/ketone + formyl‐CoA	E493	Q128	P0DUV9
Mycobacterial HACL	2‐hydroxyacyl‐CoA → aldehyde/ketone + formyl‐CoA	E472	Q120	P66947
Human HACL1	2‐hydroxyacyl‐CoA → aldehyde/ketone + formyl‐CoA	N.A.^a^	Q123	Q9UJ83
Human HACL2	2‐hydroxyacyl‐CoA → aldehyde/ketone + formyl‐CoA	I554	Q161	A1L0T0
RuHACL (bacterial)	2‐hydroxyacyl‐CoA → aldehyde/ketone + formyl‐CoA	N.A.	Q113	A0A1H8YFL8
ApbHACLs (bacterial)	2‐hydroxyacyl‐CoA → aldehyde/ketone + formyl‐CoA	N.A.	Q117	A0A3C0TX30
Oxalyl‐CoA decarboxylase (OXC)	Oxalyl‐CoA + H^+^ → CO_2_ + formyl‐CoA	N.A.	E121	P40149
Bacterial pyruvate decarboxylase (PDC)	Pyruvate + H^+^ → CO_2_ + acetaldehyde	E473	H114	P06672
Yeast PDC	Pyruvate + H^+^ → CO_2_ + acetaldehyde	E477	H115	P06169
Indolpyruvate decarboxylase	2‐oxoacid + H^+^ → CO_2_ + aldehyde	E468	H116	P23234
Yeast 2‐oxoacid decarboxylase	2‐keto‐acid (from transaminated aa) + H^+^ → CO_2_ + aldehyde	E545	H145	Q06408
Benzoylformate decarboxylase	2‐oxoacid + H^+^ → CO_2_ + aldehyde	L461	L110	P20906
Phenylpyruvate decarboxylase	2‐oxoacid + H^+^ → CO_2_ + aldehyde	L462	H113	P51852
Benzaldehyde lyase	Benzoin → 2 benzaldehyde	T481	Q113	P51853
Sulfoacetaldehyde acetyltransferase (SAAT)	Sulfoacetaldehyde + hydrogenphosphate → acetyl‐P + hydrogensulfite	E510	Q125	Q84H41
*N* ^*2*^‐(2‐carboxyethyl)‐arginine synthase	D‐glyceraldehyde‐3‐P + L‐arginine → *N* ^2^‐(2‐carboxyethyl)‐L‐arginine + hydrogenphosphate + H^+^	I496	Q121	Q9LCV9
cyclohexane‐1,2‐dione hydrolase	Cyclohexane‐1,2‐dione + H_2_O → 6‐oxohexanoate + H^+^	N484	Q116	P0CH62
**Without on‐pathway protonation of C2α‐carbanion/deprotonation of its conjugate acid**				
Pyruvate oxidase (POX)	Pyruvate + hydrogenphosphate + O_2_ + H^+^ → CO_2_ + acetyl‐P + H_2_O_2_	I480	Q122	P37063
Bacterial aceto‐2‐hydroxyacid synthase (AHAS)	Pyruvate (donor) + pyruvate (acceptor) + H^+^ → CO_2_ + acetolactate	V483	Q123	Q04789
Yeast AHAS	Pyruvate (donor) + pyruvate (acceptor) + H^+^ → CO_2_ + acetolactate	V583	Q202	P07342
Glyoxylate carboligase	Glyoxylate (donor) + glyoxylate (acceptor) + H^+^ → CO_2_ + 2‐hydroxy‐3‐oxopropanoate	I479	Q115	P0AEP7
Alpha‐hydroxy‐beta‐ketoacid synthase	2‐oxoacid (donor) + 2‐oxoacid (acceptor) + H^+^ → CO_2_ + 2‐hydroxy‐3‐oxoacid	V485	Q117	A0ABF7PQ46

^a^ N. A., not applicable, α‐helix homologous to AcHACL α‐helix with position 493 not present.

We have recently characterized actinobacterial 2‐hydroxyacyl‐CoA lyase (AcHACL) [8], another POX family enzyme catalyzing a reaction where the C2α‐carbanion intermediate needs protonation (Fig. 1). In line with its postulated role in proton transfer to the C2α‐carbanion, AcHACL possesses a PDC E473/E477 homologous Glu (E493, UniProt ID: P0DUV9). And as in PDC, mutations of this residue to Gln and Ala slow the on‐pathway cleavage of 2‐hydroxyisobutyryl (2‐HIB)‐CoA to acetone and formyl‐CoA [9]. A systematic inspection of the active site α‐helix close to the C‐terminal domain in the POX family (Table 1) revealed that the Glu is also present in a putative mycobacterial HACL (E472, UniProt ID: P66947) and in sulfoacetaldehyde acetyltransferase (SAAT; E510, UniProt ID: Q84H41). Due to the higher sequence similarity with AcHACL, it can be assumed that the uncharacterized mycobacterial enzyme also catalyzes the cleavage of short‐chain 2‐hydroxyacyl‐CoAs via protonation of the C2α‐carbanion. In contrast, the SAAT‐catalyzed reaction is more complex, as it proceeds via the elimination of sulfite from sulfoacetaldehyde and subsequent nucleophilic attack by phosphate that finally leads to acetyl‐P [10]. However, besides the elucidation of the reaction stoichiometry, SAAT has not been further characterized.

To better understand the role of the C2α‐carbanion‐protonating Glu in the POX family, we determined the AcHACL structure (wild‐type, WT, and E493A mutant) in complex with the inactive cofactor 3‐deazathiamine diphosphate (dzThDP) and 2‐methylglyceryl‐CoA, an off‐pathway carboligation product from 2‐HIB‐CoA and acetol (Fig. 1). As already demonstrated for the complex with 2‐HIB‐CoA [8], the position of the E493 side‐chain carboxylate is taken over by a well‐coordinated water molecule in the E493A mutant. Substitution of Ala by the more polar Ser at position 493 did not change kinetics for 2‐HIB‐CoA cleavage. In both mutants, WT *K*_M_ values for 2‐HIB‐CoA are almost sixfold improved. However, WT kinetics for the conversion of 2‐hydroxy‐2‐methylbutyryl‐CoA were comparable to the values previously obtained with 2‐HIB‐CoA, indicating that E493‐mediated catalysis is not affected by slightly larger substrates (C5 acyl residue versus C4 in 2‐HIB). Nevertheless, off‐pathway carboligation kinetics confirmed the improved substrate accommodation already observed for E493 mutants in the 2‐HIB‐CoA cleavage to formyl‐CoA and acetone [8]. In both on‐pathway 2‐hydroxyacyl‐CoA cleavage and off‐pathway carboligation, however, rates were substantially reduced compared to the WT enzyme, underscoring the importance of E493 in AcHACL for enabling an efficient catalysis.

## Materials and methods

### Purification of recombinant proteins

WT AcHACL and mutants were expressed from pASG‐IBA43 (IBA Lifesciences, Göttingen, Germany) in *Escherichia coli* Lemo21 (DE3) (NEB) cultivated in Terrific Broth [8]. The proteins were purified after immobilized metal affinity chromatography by size‐exclusion chromatography using a HiLoad 16/600 Superdex 200 pg column equilibrated in 20 mm Tris pH 7.5, 150 mm NaCl, 3 mm β‐mercaptoethanol. For activity assays and cryostocks, the purified proteins were transferred into conservation buffer (100 mm potassium phosphate, 10% glycerol, pH 7.2 supplemented with 1 mm ThDP and 5 mm MgCl_2_) with PD‐10 desalting columns (Cytiva, Marlborough, MA, USA). The E493S and E493D mutant enzymes were obtained by site‐directed mutations as previously described for the E493A mutant [8].

### Synthesis of 2‐hydroxyacyl‐CoAs and dzThDP

2‐HIB‐CoA, 2‐hydroxy‐2‐methylbutyryl‐CoA, and 2‐methylglyceryl‐CoA were synthesized via the corresponding thiophenol thioester intermediates [11] and stored as aqueous solutions at −20 °C. The dzThDP was synthesized from the corresponding deazathiamine via phosphorylation [8]. 2‐HIB thiophenol thioester was isolated as a white solid with 49% yield (0.524 g). ^1^H NMR (400 MHz, CDCl_3_), δ [ppm]: 7.43 (s, 5H), 2.76 (br s, 1H), 1.53 (s, 6H); ^13^C NMR (101 MHz, CDCl_3_), δ [ppm]: 205.0, 134.7, 134.7, 129.3, 129.2, 129.2, 127.6, 79.0, 27.5, 27.5. High‐resolution mass spectrometry (electrospray ionization) m/z: calculated for: C_25_H_43_N_7_O_18_P_3_S^+^ [M + H]^+^ 854.1593; found: 854.1592. 2‐Methylglyceryl thiophenol thioester was isolated as a white solid in 42% yield (0.318 g). ^1^H NMR (400 MHz, CDCl_3_), δ [ppm]: 7.43 (s, 5H), 4.04 (d, *J* = 11.1 Hz, 1H), 3.63 (br s, 1H), 3.56 (d, *J* = 11.2 Hz, 1H), 2.19 (br s, 1H), 1.44 (s, 3H); ^13^C NMR (101 MHz, CDCl_3_), δ [ppm]: 204.7, 134.7, 134.7, 129.5, 129.2, 129.2, 127.4, 81.6, 68.2, 22.3. High‐resolution mass spectrometry (electrospray ionization) m/z: calculated for: C_25_H_43_N_7_O_19_P_3_S^+^ [M + H]^+^ 870.1542; found: 870.1540.

### AcHACL activity assays

Kinetics for the 2‐hydroxyacyl‐CoA lyase and the off‐pathway carboligation reactions were determined with the purified enzymes incubated in conservation buffer at optimal temperature and pH of 37 and 7.2 °C, respectively, using a discontinuous HPLC‐based assay [8]. For the enzymatic activity, a detection limit of 0.1 nmol·min^−1^·mg^−1^ was achieved. Kinetic parameters were calculated by nonlinear regression analysis applying the Michaelis–Menten equation using graphpad prism (GraphPad Software Inc, San Diego, CA, USA).

### Crystallization and structure determination

Purified proteins were concentrated to 10 mg·mL^−1^. The already published AcHACL crystallization condition (25% PEG 1500, 0.1 m MIB buffer pH 7.0) [8] was used to set up hanging drop plates of WT and mutant AcHACL in the presence of 5 mm dzThDP, 5 mm MgCl_2_, and 1 mm of either 2‐HIB‐CoA or 2‐methylglyceryl‐CoA. All crystals were cryoprotected in crystallization buffer containing 25% glycerol and flash frozen in liquid nitrogen. Diffraction data were collected at beamline I03 at the Diamond Light Source (Didcot, Oxfordshire, UK). The structures were solved by molecular replacement with MolRep [12], using PDB entry 7PT1. Model building was performed in coot [13] and the structures were refined with Refmac5 [14]. MolProbity [15] was used to evaluate the final models and pymol (Schrödinger, LLC, New York City, NY, USA) for protein model visualization.

## Results

### AcHACL E493A and E493S mutants display similar kinetics

Previously, we reported kinetic parameters for the on‐pathway cleavage of 2‐HIB‐CoA with WT AcHACL and E493A mutant [8]. The 12‐fold higher WT *k*_cat_ (Table 2) clearly demonstrates that E493 is needed as an acid/base catalyst for an efficient proton transfer from the substrate acyl‐Cβ‐OH to the C2α‐carbanion (Fig. 1). The active site water molecule found in the mutant structure (PDB ID: 7PT3) at the position of the WT E493 side‐chain carboxylate can only partially compensate for this role. Interestingly, the E493A *K*_M_ for 2‐HIB‐CoA is about sixfold better than the WT value. The impeded accommodation of 2‐HIB in the presence of E493 (PDB ID: 7PT1) and also Q493 (PDB ID: 7PT2) is caused by an unfavorably close binding of one 2‐HIB methyl group (*Cγ*) to the E/Q493 side chain [8]. To test whether a small but more polar residue at position 493 can improve catalytic efficiency, for example, by attracting additional water molecules for proton transfer, kinetics for the 2‐HIB‐CoA cleavage were also obtained for the E493S mutant (Table 2). However, values were almost identical for the E493A and E493S enzymes, indicating that catalytically competent substrate binding is comparable and that not only Ala but also Ser is inefficient to substitute WT E493 as an acid/base catalyst. Additionally, we analyzed the more conserved E493D mutation. In line with results already obtained with corresponding bacterial [16, 17] and yeast [18] PDC mutants, the AcHACL E493D enzyme shows a dramatic loss of activity for 2‐HIB‐CoA cleavage, disqualifying this mutation for improving substrate accommodation. This finding is somewhat surprising due to similar properties of Glu and Asp carboxylate side chains. Nevertheless, it underlines the similar roles of AcHACL E493 and the homologous PDC residue in proton transfer (Fig. 1) that cannot easily be substituted by other residues.

**Table 2:** Kinetic parameters of WT AcHACL and E493 mutant enzymes obtained at optimal pH 7.2 and 37 °C for the on‐pathway cleavage of 2‐hydroxyacyl‐CoAs to formyl‐CoA and the respective ketone as well as for the off‐pathway carboligation from 2‐HIB‐CoA and a carbonyl compound substrate.

Enzyme variant	2‐hydroxyacyl‐CoA substrate	*K*_M_ (μm)	*V*_max_ (nmol^−1^·min^−1^·mg^−1^)	*k*_cat_ (s^−1^)	*k*_cat_/*K*_M_ (s^−1^·mm ^−1^)	References
**On pathway lyase reaction: 2‐hydroxyacyl‐CoA → formyl‐CoA + ketone**						
WT	2‐HIB‐CoA	120 ± 12	1200 ± 20	1.3 ± 0.04	11 ± 1	[8]
E493A	2‐HIB‐CoA	21 ± 4	98 ± 4	0.11 ± 0.004	5.1 ± 0.9	[8]
E493Q	2‐HIB‐CoA	79 ± 7	25 ± 1	0.03 ± 0.001	0.34 ± 0.03	[8]
E493S	2‐HIB‐CoA	22 ± 3	108 ± 3	0.12 ± 0.003	5.4 ± 0.8	This study
E493D	2‐HIB‐CoA	123 ± 56	2.2 ± 0.3	0.0024 ± 0.0003	0.02 ± 0.01	This study
WT	2,2‐HMB‐CoA^a^	52 ± 6	795 ± 22	0.87 ± 0.03	17 ± 4	This study

Enzyme variant	Carbonyl compound	*K*_M_ (mm)	*V*_max_ (nmol^−1^·min^−1^·mg^−1^)	*k*_cat_ (s^−1^)	*k*_ca*t*_/*K*_M_ (s^−1^·mm ^−1^)	
**Off‐pathway carboligation** ^b^ **: 2‐HIB‐CoA + carbonyl compound → 2‐hydroxyacyl‐CoA + acetone**						
WT	Acetol	37 ± 3	6070 ± 120	6.6 ± 0.1	0.18 ± 0.01	This study
E493A	Acetol	2.3 ± 0.3	320 ± 10	0.35 ± 0.01	0.15 ± 0.02	This study
E493S	Acetol	2.6 ± 0.2	474 ± 11	0.52 ± 0.01	0.20 ± 0.02	This study
E493S	Glycolaldehyde	0.84 ± 0.10	477 ± 11	0.52 ± 0.01	0.62 ± 0.07	This study
E493S	Dihydroxyacetone	4.4 ± 0.6	577 ± 18	0.63 ± 0.02	0.14 ± 0.02	This study
E493S	Pentan‐3‐one	4.8 ± 0.7	229 ± 9	0.25 ± 0.01	0.052 ± 0.008	This study

^a^ 2,2‐HMB‐CoA, racemic mixture of 2‐hydroxy‐2‐methylbutyryl‐CoAs.

^b^ Off‐pathway carboligation at excess 2‐HIB‐CoA (for assays with WT ≥ 950 μm, for E493A and E493S ≥ 400 μm).

Although WT 2‐HIB‐CoA binding seems to be impeded compared to E493A and E493S mutants, the WT *K*_M_ value for the slightly larger 2‐hydroxy‐2‐methylbutyryl‐CoA (C5 acyl residue versus C4 in 2‐HIB) is twofold better, while the *k*_cat_ value is still at 70% of the WT 2‐HIB‐CoA cleavage activity. Assuming a conserved binding mode with orientation of the acyl‐Cβ‐OH toward G43, H44, T88, and E493 [8], this enhanced binding at the active site might be mediated by improved Van‐de‐Waals interactions of the larger 2‐methylbutyryl skeleton, possibly with active side residues L127, L566, and V569.

**Fig. 3:**
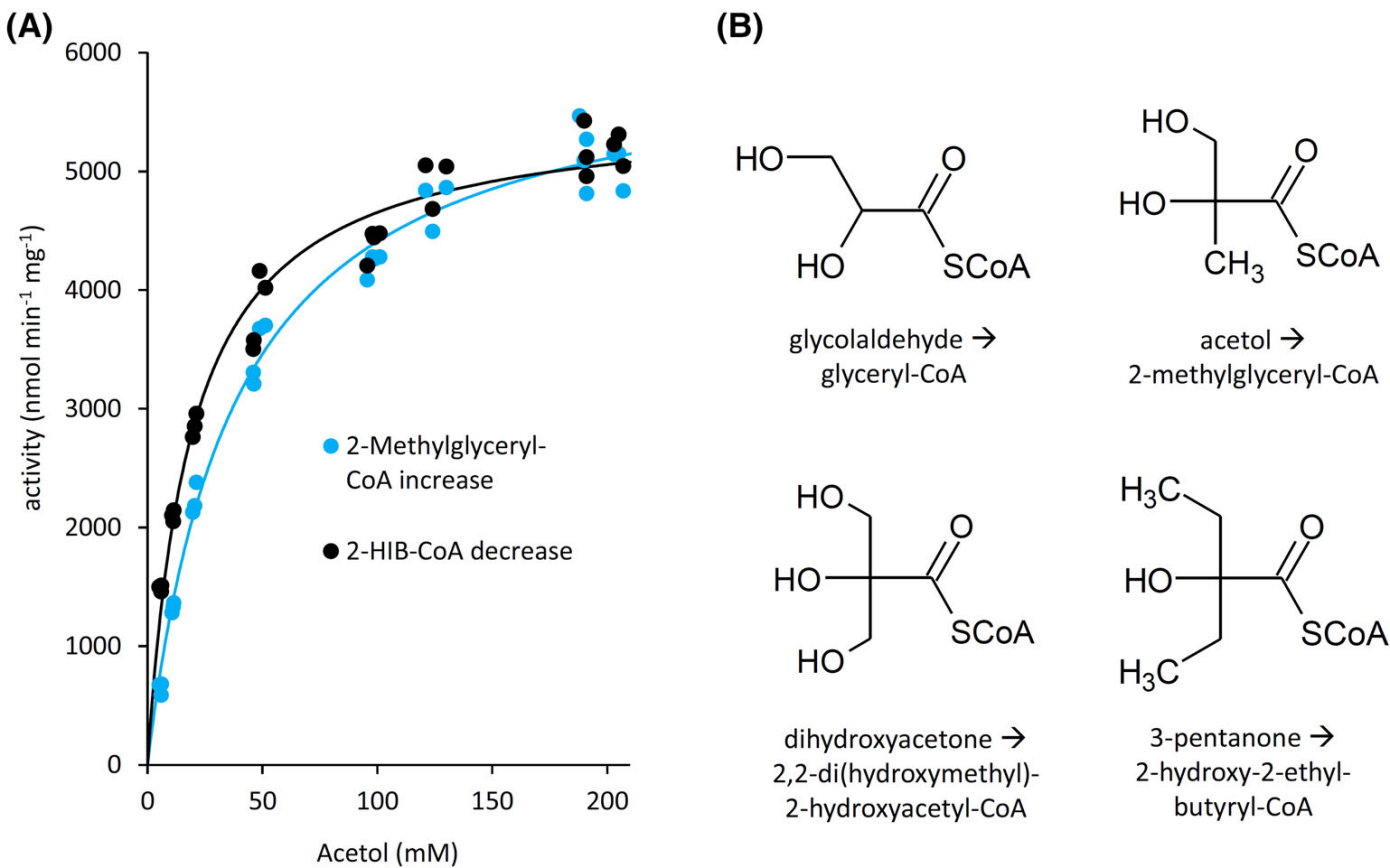
Off‐pathway carboligation proceeding via 2‐HIB‐CoA cleavage and reaction of the resulting C2α‐carbanion with various carbonyl compounds. (A) Kinetic plot for AcHACL WT incubated with excess 2‐HIB‐CoA and acetol at indicated concentrations. At a concentration of about two to three times *K*_M_ for acetol, the carboligation dominates and solely 2‐methylglyceryl‐CoA is formed. At lower acetol concentrations, however, formation of formyl‐CoA from the complete lyase reaction is detected as well. (B) 2‐Hydroxyacyl‐CoA products of the AcHACL E493S mutant‐catalyzed carboligation reactions kinetically characterized in this study.

Off‐pathway carboligation kinetics for the formation of 2‐methylglyceryl‐CoA from 2‐HIB‐CoA and acetol was studied with WT AcHACL as well as with E493A and E493S mutants (Table 2, Fig. 3). Compared to the on‐pathway cleavage of 2‐HIB‐CoA to formyl‐CoA, *k*_cat_ of the carboligation is three‐ to fivefold increased. In conclusion, the initial formation of the C2α‐carbanion from 2‐HIB‐CoA and the subsequent off‐pathway carboligation to 2‐methylglyceryl‐CoA (Fig. 1) are much faster than the on‐pathway release of formyl‐CoA. Likely, the protonation of the C2α‐carbanion prior to the release of formyl‐CoA represents the rate‐determining step in the on‐pathway reaction. Furthermore, the reduced rates obtained with the E493 mutants, being > 10 times lower than the WT value, point to a crucial role of E493 as acid/base catalyst also in the off‐pathway carboligation step. However, as the carboligation assay cannot distinguish between the rates of the initial deprotonation of the 2‐HIB‐Cβ‐OH and the off‐pathway protonation of the carbonyl‐O atom (Fig. 1), the effect of E493‐mediated catalysis on the carboligation rate cannot be quantified. Although E493 guarantees an efficient carboligation catalysis, catalytically competent substrate accommodation is again impeded, as WT *K*_M_ of about 40 mm for acetol is reduced to values below 3 mm with the mutants.

### E493S *K*_M_ values for short‐chain carbonyl compounds vary between 1 and 5 m m

To avoid the chemical preparation of additional 2‐hydroxyacyl‐CoAs or the instable formyl‐CoA, which are required as substrates for the AcHACL‐catalyzed on‐pathway lyase and synthase reactions, respectively, the enzyme's potential for synthesis of various 2‐hydroxycarboxylic acids was tested employing the off‐pathway carboligation reaction. Since the WT turned out to be unsuitable for these assays due to high *K*_M_ values for carbonyl compounds (Fig. 3A), we used the E493S mutant for further characterization. Besides for acetol, kinetic parameters were obtained for glycolaldehyde, dihydroxyacetone, and pentan‐3‐one (Table 2, Fig. 3B). With glycolaldehyde (C2 compound) the lowest *K*_M_ value of about 0.8 mm was achieved. More than 5‐times higher values were obtained with dihydroxyacetone and pentan‐3‐one. Despite having carbon skeletons of different lengths, *K*_M_ values for the polar dihydroxyacetone (C3 compound) and the non‐polar pentan‐3‐one (C5 compound) are quite similar. This indicates that not additional polar or non‐polar groups but rather the total number of O and C atoms in the molecule seems to be crucial for active site substrate binding. Accordingly, product formation with 2‐methylpentan‐3‐one tested at concentrations of 5–20 mm was detectable with both the E493A and E493S mutants, albeit at very low turnover rates not allowing determination of kinetic parameters. Finally, incubation with 2,4‐dimethylpentan‐3‐one at concentrations of 5–20 mm produced no carboligation product, suggesting that the molecule is already too large to fit into the active site.

In contrast to *K*_M_, the obtained *k*_cat_ value with the E493S mutant appears to depend more on the polarity of the carboligating substrate. While glycolaldehyde and acetol with one hydroxyl group display similar rates, *k*_cat_ increases 20% with dihydroxyacetone. In agreement with this trend, the *k*_cat_ value for the carboligation with pentan‐3‐one is only 50% of the value obtained for acetol and glycolaldehyde. Possibly, the lack of hydroxyl groups in pentan‐3‐one reduces the number of water molecules at the active site, thus reducing the efficiency of the carbonyl‐O atom protonation needed in the carboligating step.

### WT and E493 mutant structures with 2‐HIB‐CoA are almost identical

**Fig. 4:**
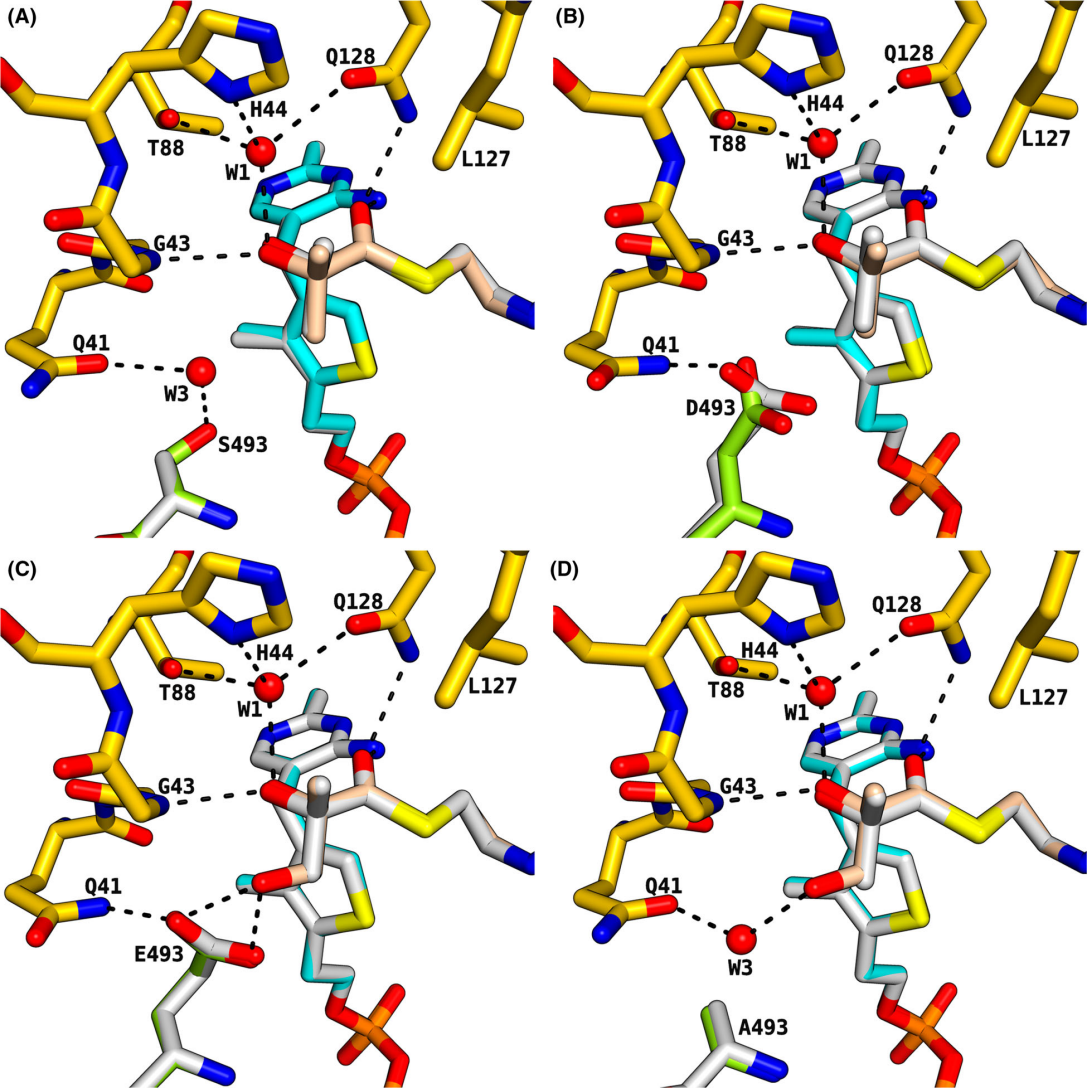
Structural superposition of active sites of AcHACL WT and various mutants trapped with inactive cofactor dzThDP and non‐covalently bound 2‐hydroxyacyl‐CoAs. The active site is located between two protein chains, as shown by the different colors of the amino acids (green and yellow). The inactive cofactor dzThDP (cyan) was used to obtain 2‐hydroxyacyl‐CoA‐bound structures. (A) The E493S mutant with 2‐HIB‐CoA (beige, PDB ID: 9QZ5) is superimposed with the E493A mutant structure (gray, PDB ID: 7PT3). (B) The E493D mutant with 2‐HIB‐CoA (beige, PBD ID: 9QZ4) is superimposed with the WT structure (gray, PDB ID: 7PT1). (C) WT structures with bound (*s*)‐2‐methylglyceryl‐CoA (beige, PDB ID: 9QZ6) and 2‐HIB‐CoA (gray, PDB ID: 7PT1). (D) E493A mutant structures with bound (*s*)‐2‐methylglyceryl‐CoA (beige, PDB ID: 9QZ7) and 2‐HIB‐CoA (gray, PDB ID: 7PT1). W1 and W3 represent active site water molecules. Dashed black lines indicate H‐bonds.

The AcHACL E493D and E493S mutant structures were solved with dzThDP and 2‐HIB‐CoA to a resolution of 1.63 Å and 1.57 Å, respectively (Table 3). As shown previously for WT and E493A enzymes [8], these structures are good approximations to the Michaelis complex, as the cofactor C2 atom is positioned in a distance of about 3 Å to the 2‐HIB carbonyl‐C atom. In line with almost identical kinetic parameters for E493A and E493S mutants (Table 2), the E493S structure in complex with dzThDP and 2‐HIB‐CoA does not deviate much from the corresponding E493A structure (Fig. 4A). Once again, the position of WT E493 side‐chain carboxylate is occupied by a water molecule (W3 in Fig. 4A). Consequently, 2‐HIB methyl groups are now well‐accommodated, compared to the suboptimal situation in the WT [8]. Important interactions with other active site residues, particularly with Q128, are not affected by the mutation at position 493. As shown previously for AcHACL [8], the interaction of the substrate acyl carbonyl‐O atom with Q128 is accompanied with H‐bonding to the aminopyrimidine‐N4′ atom (Fig. 4A). These interactions guarantee the catalytically competent orientation of the substrate molecule and enable proton transfer from N4′ to the carbonyl‐O atom. They are maintained during the formation of the tetrahedral intermediate, which is covalently bound to the thiazolium C2 atom. The subsequently breaking bond is directed perpendicular to the ring plane [19] in a “maximum orbital overlap” conformation (the scissile bond for the C2α‐carbanion formation shown in Fig. 1 is the C2α‐R_2_ bond in the forward and the C2α‐H bond in the reverse reaction). Accordingly, Q128 is conserved in many ThDP‐dependent enzymes, for example, in all HACL, SAAT, AHAS, and POX enzymes (Table 1).

**Table 3:** Crystallographic data and refinement statistics.

	AcHACL_E493D + dzThDP + 2‐HIB‐CoA	AcHACL_E493S + dzThDP + 2‐HIB‐CoA	AcHACL_WT + dzThDP + 2‐methylglyceryl‐CoA	AcHACL_E493A + dzThDP + 2‐methylglyceryl‐CoA
Data collection				
Beamline	DLS I03	DLS I03	DLS I03	DLS I03
Wavelength (Å)	0.9763	0.9763	0.9762	0.9762
Space group	C222_1_	C222_1_	C222_1_	C222_1_
Cell dimensions				
*a*, *b*, *c* (Å)	104.0/147.5/174.5	103.9/148.2/174.6	103.8/146.5/174.3	103.9/145.8/174.3
*α*, *β*, *γ* (°)	90.0/90.0/90.0	90.0/90.0/90.0	90.0/90.0/90.0	90.0/90.0/90.0
Resolution (Å)	87.23–1.63 (1.83–1.63)^a^	87.29–1.57 (1.74–1.57)^a^	84.68–1.55 (1.69–1.55)^a^	87.17–1.72 (1.89–1.72)^a^
*R*_meas_ [%]	13.0 (166.9)	9.9 (153.8)	10.2 (168.2)	19.3 (190.8)
< *I*/*σI*>	12.7 (1.7)	16.3 (1.8)	18.7 (1.6)	11.2 (1.6)
Completeness (%)	94.7 (67.4)^b^	93.9 (59.2)^b^	94.0 (72.2)^b^	94.0 (71.8)^b^
Redundancy	11.6 (11.4)	11.5 (11.9)	13.8 (13.3)	13.5 (12.9)
CC(1/2)	1.0 (0.7)	1.0 (0.7)	1.0 (0.7)	1.0 (0.6)
Refinement				
*R*_work_/*R*_free_ [%]	15.7/19.3	16.4/20.1	15.8/18.4	16.2/20.4
Ramachandran plot				
Most favored [%]	97.7	97.2	97.5	97.1
Allowed [%]	2.3	2.8	2.5	2.9
Disallowed [%]	0.0	0.0	0.0	0.0
No. atoms				
Protein	8609	8656	8643	8633
Ligands	164	163	165	165
Water	991	918	1065	1043
*B*‐factors				
Protein	18.6	21.9	16.6	19.5
Ligands	17.2	21.8	18.5	21.8
Water	30.4	28.4	31.3	33.4
R.m.s. deviations				
Bond lengths (Å)	0.0078	0.0076	0.0088	0.0068
Bond angles (°)	1.6730	1.6760	1.7890	1.6160
PDB‐ID	9QZ4	9QZ5	9QZ6	9QZ7

^a^ Values in parentheses are for the highest resolution shell

^b^ Ellipsoidal completeness.

In stark contrast to the kinetic data (Table 2), WT and E493D Michaelis complexes with 2‐HIB‐CoA are almost identical (Fig. 4B). Due to the bending of the larger Glu side‐chain, one D493 β‐carboxylate‐O atom is even slightly closer to the 2‐HIB‐Cβ‐O atom (3.9 Å compared to WT between 4.0 Å and 4.1 Å). Accordingly, the distance between the other side chain carboxylate‐O atom and a 2‐HIB‐methyl‐C atom is still unfavorable (3.2 Å compared to WT 3.1 Å). Among the other interactions of the side‐chain carboxylate at position 493, the H‐bond to Q41 is maintained in the mutant. However, the H‐bond to the active site water connecting E493 with D496 in the WT enzyme is disrupted (not shown in Fig. 4). It remains to be elucidated whether these small changes can explain the > 500‐fold activity reduction in the E493D mutant. In this connection, the roles of AcHACL Q41 and D496 have not yet been characterized.

### WT and E493A AcHACL bind only (*s*)‐2‐methylglyceryl‐CoA

The AcHACL WT and E493A mutant structures were solved with dzThDP and 2‐methylglyceryl‐CoA to a resolution of 1.55 Å and 1.72 Å, respectively (Table 3). The overall structures are identical to the previously determined ones solved with 2‐HIB‐CoA (PDB ID: 7PT1 and 7PT3), demonstrating a conserved binding mode for the cofactor and CoA residue in the presence of different acyl thioesters. Accordingly, the specific interactions already seen for 2‐HIB [8] are also present in the WT (Fig. 4C) and E493A mutant (Fig. 4D) structures with 2‐methylglyceryl‐CoA. The carbonyl‐C atom of the acyl residue (Cα in Fig. 1) is in close proximity to the thiazolium C2 atom (about 3.3 Å). This positioning is achieved through H‐bond interactions of the acyl‐Cα‐O atom with N4′ of the aminopyrimidine ring and the Q128 side chain. Furthermore, the acyl‐Cβ‐OH interacts with G43 and a central water (W1 in Fig. 4) that is well‐coordinated by H‐bonding with H44, T88, and Q128. Although the enzymes were co‐crystallized with the racemic mixture of 2‐methylglyceryl‐CoA, only the (*s*)‐enantiomer was found in the structures. Consequently, the acyl‐Cγ‐OH (not present in the 2‐HIB residue) is oriented toward WT E493 and forms H‐bonds with its side‐chain carboxylate. The suboptimal distance (3 Å) between the acyl‐Cγ atom and the E493 side‐chain carboxylate observed with 2‐HIB‐CoA is conserved in the structure with (*s*)‐2‐methylglyceryl‐CoA. Due to the strong interactions between the acyl‐Cα‐O atom and Cβ‐OH at the active site, the only option for the acyl‐Cγ‐OH in the (*R*)‐enantiomer would be orientation toward residues L127, L566, and V569. Although this positioning might enable H‐bonding with the central W1, it is clearly disfavored due to the otherwise predominantly hydrophobic environment. A similar situation is found in the E493A mutant. The WT position of the side‐chain carboxylate is occupied by a water molecule (W3) that mimics the role of E493 in the interaction with the acyl‐Cγ‐OH of (*s*)‐2‐methylglyceryl‐CoA. As previously shown in the mutant structure with 2‐HIB‐CoA, this water is also well coordinated with H‐bonds to another water and to Q41.

## Discussion

AcHACL catalyzes the reversible cleavage of 2‐hydroxyacyl‐CoAs to formyl‐CoA and corresponding aldehydes or ketones. The importance of the WT E493 in the proton transfer from the 2‐HIB‐Cβ‐OH to the C2α‐carbanion intermediate during 2‐HIB‐CoA cleavage to acetone and formyl‐CoA was previously established [8]. Here, we now provide kinetic information on other substrates as well as structural information on the Michaelis complex with (*s*)‐2‐methylglyceryl‐CoA. Consistently, the replacement of E493 with Ala or Ser is accompanied with reduced *k*_cat_ but improved *K*_M_. The trade‐offs between substrate accommodation and conversion efficiency may occasionally have led to the evolution of enzymes that catalyze reactions involving C2α‐carbanion protonation, but which lack E493 homologs. Accordingly, several of these cases can be identified within the POX family (Table 1). However, the replacement of the Glu residue for improving substrate binding or enabling usage of new substrates is not a general rule and often other structural changes co‐occur.

### Roles of AcHACL E493 and homologous residues in POX family 2‐oxoacid decarboxylases

The unfavorable binding of 2‐HIB‐CoA and (*s*)‐2‐methylglyceryl‐CoA by WT AcHACL reveals that the enzyme pays the price for efficient catalysis enabled by acid/base catalyst E493 in the form of impeded substrate accommodation. However, in AcHACL, alternative residues or active site water cannot fully compensate for the multiple catalytic functions of E493. The E493D mutation appears to be most straightforward to improve substrate binding while maintaining high activity in AcHACL. However, as previously demonstrated in bacterial and yeast PDC [17, 18], the replacement of the Glu by the slightly smaller Asp is extremely detrimental for activity. This is somewhat surprising, as our WT AcHACL and E493D mutant structures with dzThDP and 2‐HIB‐CoA turned out to be almost identical. Based on a structure of bacterial PDC E473D with bound lactyl‐ThDP intermediate (PDB ID: 3OE1), the activity loss found in the aspartate mutant was explained by stereoelectronic effects resulting in the deviation of the scissile bond for decarboxylation from the optimal perpendicular orientation by ~ 20° to 30° [17]. However, a closer inspection of the data revealed that the electron density is insufficient for the postulated lactyl‐ThDP ligand with the deviating bond angle. Hence, stereoelectronic effects caused by the PDC E473D mutation have not been demonstrated thus far. In conclusion, the substantial loss of catalytic efficiency caused by the Glu‐to‐Asp exchange remains enigmatic in AcHACL as well as in PDC. In both enzymes, the Glu interacts with the substrate via the leaving group (*R*_2_ in Fig. 1) and is important for C2α‐carbanion protonation. Additionally, the AcHACL E493 is involved in the deprotonation of the acyl‐Cβ‐OH in the initial cleavage step and, correspondingly, the protonation of the carbonyl‐O atom of aldehydes and ketones in the synthase and off‐pathway carboligation reactions. Obviously, these multiple roles of the Glu in AcHACL and PDC are not compatible with Asp. On the other hand, mutations with the smaller residues Ala and Ser could at least improve substrate binding in AcHACL. Accordingly, replacement of the Glu is also observed within the POX family decarboxylases.

In the subgroup of enzymes that uniformly catalyze the decarboxylation of 2‐oxoacids to the corresponding aldehydes, the Glu residue is only strictly conserved in the pyruvate‐specific enzymes, for example, in bacterial and yeast PDC. More diversity can be found in the decarboxylases capable of using aromatic 2‐oxoacids. The Glu is conserved in the indolepyruvate‐using decarboxylase from *Enterobacter cloacae* (E468, UniProt ID: P23234). Compared to bacterial PDC, the binding pocket is mainly enlarged due to the replacement of two Trp by smaller residues [20]. Similar changes plus the replacement of the Glu by Leu can be found in related enzymes, for example, in the phenylpyruvate decarboxylase from *Azospirillum brasilense* (L462, UniProt ID: P51852). While the Leu contributes to catalysis by decreasing the dielectric constant at the active site [21], the required acid/base catalysis is likely taken over by a D25‐H112‐D282 triad [22]. This triad is conserved in bacterial PDC additionally bearing the E493 homolog. Consequently, due to multiple catalytic residues, the proton transfer from solvent in 2‐oxoacid decarboxylases may more easily compensate for the loss of one catalytic residue than the more specific transfer from the acyl‐Cβ‐OH in AcHACL (Fig. 1). In line with this, the Asp‐His‐Asp triad found in the phenylpyruvate decarboxylase and in PDCs is not conserved in the E493 homolog‐lacking benzoylformate decarboxylase from *Pseudomonas putida*, but is replaced by a likewise complex S26‐H70‐H281 triad [23].

### The HACL reaction does not proceed by a uniform mechanism

**Fig. 5:**
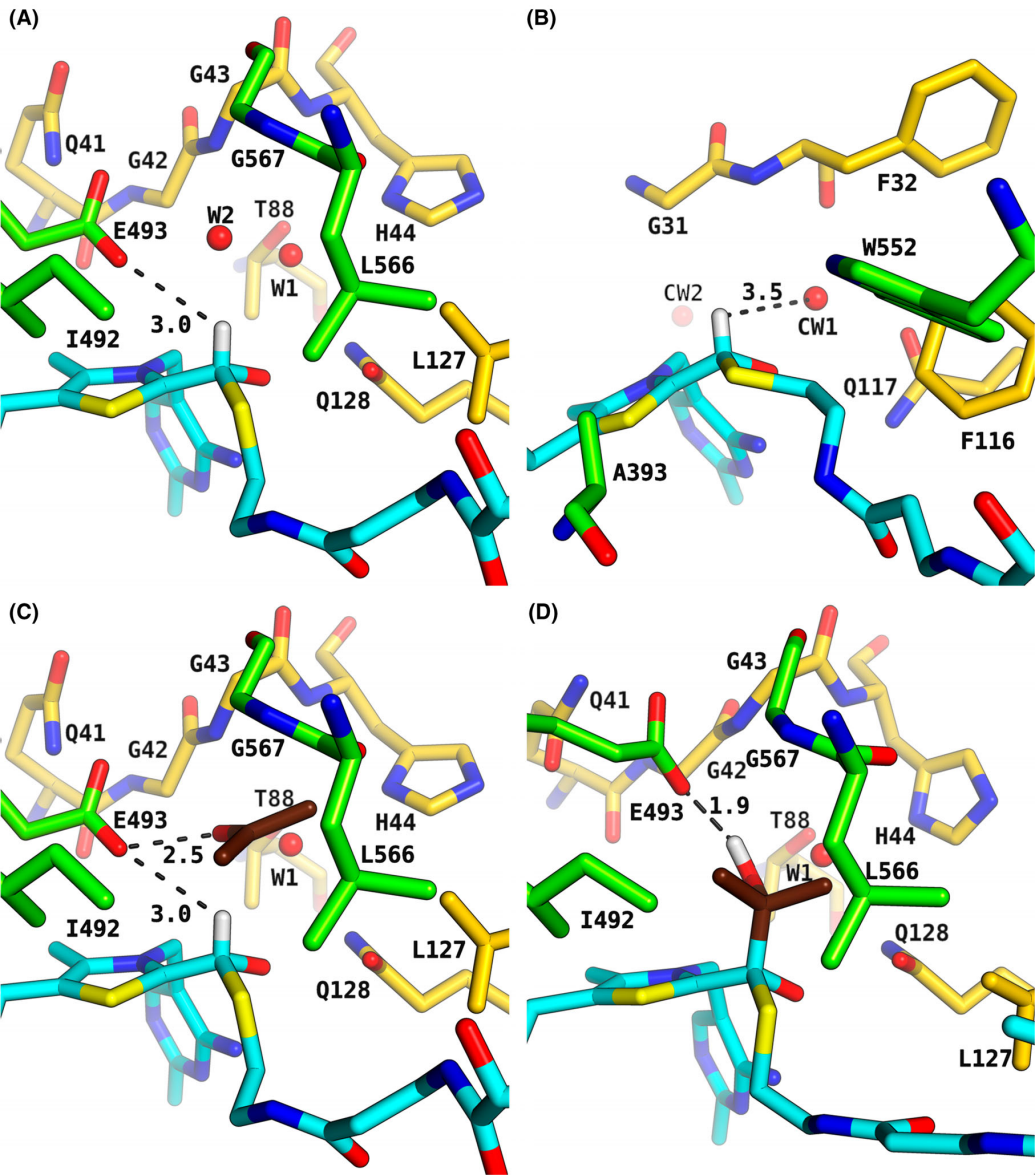
Glutamate‐dependent and glutamate‐independent protonation/deprotonation of the C2α‐carbanion/conjugate acid in HACL enzymes. (A) AcHACL active site with acid/base catalyst E493 (PDB ID: 7PT4). The interacting amino acids are colored green or yellow, depending on the protein chain. The intermediate formed by the covalent binding of the cofactor ThDP and formyl‐CoA (PDB ID: OXT) is shown in cyan. The C2α‐OH group is oriented toward the cofactor N4′ atom and the Q128 side‐chain. Due to this, the scissile bond for carbanion formation (C2α‐H bond) is positioned perpendicular above the thiazolium plane. (B) Active site of an alphaproteobacterial HACL1‐related enzyme lacking an E493 homolog (PDB ID: 8VZC, UniProt ID: A0A3C0TX30) with modeled ligand OXT in the catalytically competent binding mode shown in (A) for AcHACL. (C) The superposition of the two protein chains in AcHACL (PDB ID: 7PT4) with non‐covalently bound acetone (sepia) and OXT, respectively, illustrates the E493‐mediated reaction mechanism in AcHACL. The carbonyl compound binds directly above the C2α atom with its carbonyl‐O atom in close proximity to E493. In the carboligation/synthase reaction, E493 would transfer the proton from the C2α atom to the carbonyl‐O atom. (D) Result of a molecular dynamics simulation of AcHACL active site with the tetrahedral intermediate that would form during the cleavage of 2‐HIB‐CoA or after the attack of the C2α‐carbanion on acetone [8]. W1, W2, CW1, and CW2 represent active site water molecules. Black dashed lines indicate important H bonds (with distance given in Å).

Considering the important role of E493 in AcHACL (Fig. 5A), it is quite remarkable that several other POX family enzymes catalyzing the reversible 2‐hydroxyacyl‐CoA cleavage do not possess a homologous Glu, for example, human HACL1 (UniProt ID: Q9UJ83) of the peroxisomal α‐oxidation pathway. HACL1 catalyzes the cleavage of 2‐hydroxyphytanoyl‐CoA (C20 acyl residue) and other long‐chain substrates [24]. The HACL1 AlphaFold model [25] lacks not only the Glu residue but also the complete α‐helix, likely to enable binding of the larger thioesters. Accordingly, the large C‐terminal lid domain restricting substrate size in AcHACL [8] is only rudimentarily present in HACL1. A similar situation can be found in HACL1‐related enzymes with known crystal structure, namely, a *Rhodospirillales* enzyme (PDB ID: 6XN8) and enzymes screened for high‐rate condensation of formyl‐CoA with short‐chain aldehydes (C1 to C3) [26]. Compared to AcHACL, these HACL1 enzymes have an active site architecture with different determinants for substrate binding (Fig. 5B). The absence of the α‐helix means that at least long linear acyl residues formed from aldehydes are accommodated quite freely and are only slightly restricted by F32 and I475 (numbering as in PDB ID: 8VZC). In contrast, more restrictions can be expected for the synthesis of tertiary‐branched 2‐hydroxyacyl‐CoAs from ketones. The identified catalyst corresponding to AcHACL E493 is a water molecule (CW1 in Fig. 5B) coordinated by the conserved Q117 (AcHACL Q128) and the F32 backbone [26]. In conclusion, the proton exchange between the Cβ‐OH of 2‐hydroxyacyl‐CoAs and the C2α‐carbanion can be catalyzed efficiently by at least two different catalysts acting from different active site positions (Fig. 5).

### Use of AcHACL and HACL1 enzymes for different challenges in biotechnology

Recently, enzymatic synthesis of 2‐hydroxyacyl‐CoAs from formyl‐CoA and carbonyl compounds attracted much attention, and various screened and engineered HACLs and related oxalyl‐CoA decarboxylases (OXCs) possessing a CoA‐binding site were employed in synthetic one‐carbon assimilation routes [27]. In this context, implementation of a biotechnological process is mainly challenged by the cytotoxicity of formaldehyde needed for the catalyzed condensation with formyl‐CoA to the assimilation metabolite glyoxyl‐CoA. Hence, much focus has been laid on enzymes displaying reduced *K*_M_ for formaldehyde. Currently, the best values achieved are as low as 5 mm for an engineered OXC [28] and 2 mm for a HACL1‐related enzyme [26]. However, a second requirement seems to be high substrate specificity, as the interfering with other metabolic sequences in the production strain must be avoided. Considering the manifold options for aldehyde substrate accommodation within the relatively large active site found in the HACL1 subgroup (Fig. 5B), it might be difficult to establish sufficiently selective conditions. In line with this, catalytic efficiencies for C1 to C3 aldehydes did not show a clear trend depending on substrate size and are quite similar in these enzymes, showing maximally a 10‐fold difference [26]. More importantly, the absence of the α‐helix and large C‐terminal lid domain present in AcHACL for restricting substrate size [8] might also allow conversion of all kinds of medium‐ and long‐chain substrates in the HACL1 subgroup. Accordingly, human HACL1 catalyzes the synthesis of various aliphatic and aromatic 2‐hydroxyacyl‐CoAs from corresponding aldehydes [29].

While there is likely no simple solution for improving the specificity of short‐chain aldehydes that do not possess additional functional groups, the AcHACL structures with bound (*s*)‐2‐methylglyceryl‐CoA revealed indeed stereospecific interactions with the acyl‐Cγ‐OH. Besides its smaller size, the AcHACL active site is also more polar compared to HACL1 enzymes (Fig. 5). This feature is conserved in the E493A and E493S mutants, as additional water is attracted for maintaining specific interactions with substrates. Hence, the AcHACL structure might be a suitable start for developing enzymes with high (stereo)specificity for short‐ or even medium‐chain substrates bearing additional polar groups. In corroboration, the *K*_M_ value for glycolaldehyde is already as low as 0.8 mm in the E493S mutant. On the other side, the 5‐fold higher *K*_M_ values for dihydroxyacetone and pentan‐3‐one suggests that the active site of AcAHCL is already challenged by the presence of slightly larger substrates. Our high‐resolution structures (Fig. 5A,C,D) showing the Michaelis complex and the C2α‐carbanion/conjugate acid as well as the molecular dynamics simulation of the tetrahedral intermediate [8] can be used for rational design, for example, to improve binding of polar short‐ and medium‐chain substrates. Particularly, AcHACL appears to be a good starting point for the synthesis of tertiary‐branched 2‐hydroxyacids from short‐chain polar ketones. With this, even quite complex polyhydroxyacids could be enzymatically synthesized in one step from the corresponding carbonyl compounds. And among the simpler 2‐hydroxyacids, the green production of the poly(methyl methacrylate) precursor 2‐hydroxyisobutyrate from formyl‐CoA and acetone might be an interesting alternative to the conventional acetone cyanohydrin route [30] as well as to previously proposed biotechnological processes [31].

## Conflict of interest

The authors declare no conflict of interest.

## Author contributions

TR and MZ were involved in conceptualization; TR and MZ were involved in formal analysis; TR, MZ, BS, RL, and ZL were involved in investigation; TR and MZ were involved in methodology; MZ and TR were involved in writing and original draft.

## Data Availability

The atomic coordinates and structure factors have been deposited in the Protein Data Bank (http://wwpdb.org/) and are available under the accession codes 9QZ4 to 9QZ7.
